# Use of Fibonacci numbers in lipidomics – Enumerating various classes of fatty acids

**DOI:** 10.1038/srep39821

**Published:** 2017-01-10

**Authors:** Stefan Schuster, Maximilian Fichtner, Severin Sasso

**Affiliations:** 1 Dept. of Bioinformatics, Friedrich Schiller University, Ernst-Abbe-Platz 2, 07743 Jena, Germany; 2 Institute of General Botany and Plant Physiology, Friedrich Schiller University, Dornburger Str. 159, 07743 Jena, Germany

## Abstract

In lipid biochemistry, a fundamental question is how the potential number of fatty acids increases with their chain length. Here, we show that it grows according to the famous Fibonacci numbers when *cis*/*trans* isomerism is neglected. Since the ratio of two consecutive Fibonacci numbers tends to the Golden section, 1.618, organisms can increase fatty acid variability approximately by that factor per carbon atom invested. Moreover, we show that, under consideration of *cis*/*trans* isomerism and/or of modification by hydroxy and/or oxo groups, diversity can be described by generalized Fibonacci numbers (e.g. Pell numbers). For the sake of easy comprehension, we deliberately build the proof on the recursive definitions of these number series. Our results should be of interest for mass spectrometry, combinatorial chemistry, synthetic biology, patent applications, use of fatty acids as biomarkers and the theory of evolution. The recursive definition of Fibonacci numbers paves the way to construct all structural formulas of fatty acids in an automated way.

Fatty acids (FAs) are of crucial importance for all organisms and many viruses. They occur, for example, within triglycerides, which serve as energy and carbon stores, and within phospholipids in biomembranes^1^. Many lipids such as diacylglycerol play an important role in cellular signalling. The importance of FAs is further underlined by differences between healthy and diseased cells, so that FAs are medically relevant biomarkers. Several FAs with conjugated double bonds exert inhibitory effects on cancer cells^2^. Many fatty acids and lipids are involved in fungal development and pathogenicity^3^,^4^.

A high diversity of FAs is beneficial for regulating various properties of membranes such as fluidity^5^, for optimizing packaging within lipoproteins as well as for their signalling function between plants and plant pathogens^6^. While an enormous multitude of more than 1000 different FAs occur in living organisms, only around 20 FAs are widely found. Palmitic (16:0), oleic (18:1) and linoleic acids (18:2; Fig. 1a) account for around 80% of commodity oils and fats^7^ (numbers in parentheses indicate the numbers of carbon atoms and double bonds). There are striking differences in different kingdoms and lineages: while higher plants synthesize more than 300 different FAs, higher animals synthesize a far smaller range^8^. Despite the wealth of known FAs, it is not immediately clear whether all theoretically possible variants of a given chain length are really used in living nature because synthesizing all of them in a coordinate way would require many different enzymes.

Most natural FAs have even-numbered chain lengths up to 22 carbon atoms, while some FAs reach chain lengths of more than 30 (e. g., on plant cuticles)^7^. Even-chain FAs are commonly synthesized by condensing and reducing several two-carbon units from acetyl-coenzyme A molecules^1^. Odd-chain FAs occur in low quantities in many different species of microorganisms, plants and some animals^9^. For example, pentadecanoic acid reaches a level of approximately 1% in cow milk fat and is made by bacteria in the rumen^7^. Other examples are margaric acid (17:0), a common constituent of lipids, pelargonic acid (9:0), occurring as esters in pelargonium oil, and valeric acid (5:0), occurring in valerian^7^. Linoleic acid and α-linolenic acid (18:3) are two examples of polyunsaturated FAs (PUFAs) and are essential constituents of the human diet^1^. Less common dietary PUFAs such as eicosapentaenoic acid (20:5) are health-promoting and can be obtained from fish or algae^10^. Allenic FAs (two adjacent double bonds) and cumulenic FAs (three or more adjacent double bonds) are rare in nature due to their decreased stability^11^. At least three allenic FAs have been found in *Phlomis* (Lamiaceae)^12^. One of them is phlomic acid (20:2) with double bonds at positions 7 and 8.

Propionate and butyrate are short-chain FAs (SCFAs) produced by the microbiome in the human gut^13^. While unbranched side chains are most common, there are some examples of branched FAs such as phytanic acid, a chlorophyll catabolite^14^. Hydroxylated FAs include ricinoleic acid (Fig. 1b), cutin acids, which are the building blocks of the polymer cutin covering the aerial surfaces of plants^7^, and several dihydroxy-octadecadienoic acids (18:2) produced by the fungus *Aspergillus nidulans* and having effects on sporulation^3^.

The goal of the present study is to elucidate the combinatorial multitude of unbranched FAs when allowing for double bonds. This covers a large range of FAs. We here derive formulas for enumerating FAs in dependence on the number of carbon atoms involved. As the number of double bonds and, thus, the number of hydrogens can vary for any given chain length, the different forms are not necessarily isomers, but can be called congeners^15^. Many enumeration techniques in mathematical chemistry are focussed on isomers^16^, although some of these techniques can also be used for counting congeners^17^,^18^,^19^. In recent years, the study of congeners (which have similar structures and may or may not have different sum formulas) has attracted more and more interest^15^,^20^,^21^,^22^. For example, the congeners of common persistent organic pollutants with at most *p* different substituents instead of hydrogens were enumerated by a graph isomorphism algorithm^21^. Gutman^22^ calls the different Kekulé structures of aromatic compounds congeners. For the present analysis, we make use of the relatively simple chemical structure of unbranched FAs. We will exactly define in each case which congener classes of fatty acids we will analyse.

We derive both recursion and explicit formulas for unmodified and two classes of modified FAs. For each of these, we distinguish two cases depending on whether or not *cis*/*trans* isomerism is considered. Our analysis not only answers a fundamental question but may also support applications such as lipidomics, a high-throughput technology used for the simultaneous detection and quantification of a large number of lipid species.

## Results

**Figure 1:**
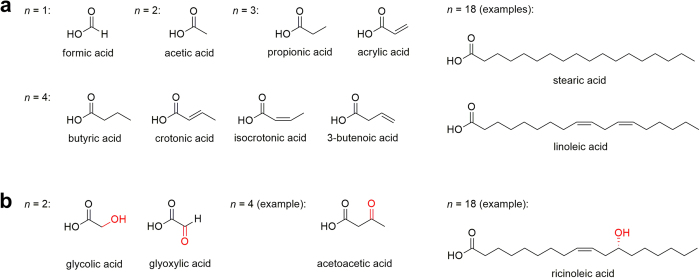
Fatty acid structures. (**a**) Unmodified fatty acids (see main text for definition). All variants are shown for *n* = 1 up to *n* = 4, whereas select examples are shown for *n* = 18. (**b**) Examples of oxidized fatty acids with modifications shown in red.

Following a broad definition that includes all chain lengths^7^, we here define FAs as straight-chain (unbranched) aliphatic monocarboxylic acids that contain carbon-carbon single or double bonds (Fig. 1a). Further below, we allow for modified FAs including hydroxy and oxo groups. For the sake of easy comprehension, we deliberately build the proof on the recursive definition of Fibonacci numbers and related series rather than on more sophisticated techniques of chemical combinatorics.

### Unmodified fatty acids with *cis*- and *trans*-isomers combined

In a first approach, we do not count *cis-* and *trans-*isomers of unsaturated FAs separately. Allenic and cumulenic FAs are first neglected as well, but will be considered in one of the classes studied below. Let *x*_*n*_ denote the number of theoretically possible, unmodified FAs involving *n* carbons. For *n* = 1, we just have the carboxy group linked to one hydrogen, which makes up formic acid (Fig. 1a). For *n* = 2, there is only one possibility to attach a methyl group to the carboxy group, giving rise to acetic acid. For *n* = 3, the saturated FA is propionic acid. However, there is also the possibility to insert a double bond, giving rise to acrylic acid (Fig. 1a). Thus,


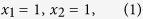


and *x*_3_ = 2. A general enumeration procedure can be derived by standard methods from discrete mathematics^23^. We can code single bonds by 0 and double bonds by 1. As no two double bonds must be adjacent to each other, we look for the number of all binary strings (consisting of 0 and 1 digits) of a given length without consecutive 1 digits. The length is *n*−2 because the carbon atom of the carboxy group cannot engage in a carbon-carbon double bond, and the remaining *n*−1 carbons are connected by *n*−2 bonds. The corresponding number series can be calculated by the recursion formula


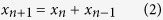


and the initial conditions (1)^23^.

Eqs. (1) and (2) define the famous series of Fibonacci numbers^23^,^24^,^25^. The series reads


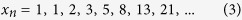


**Figure 2:**
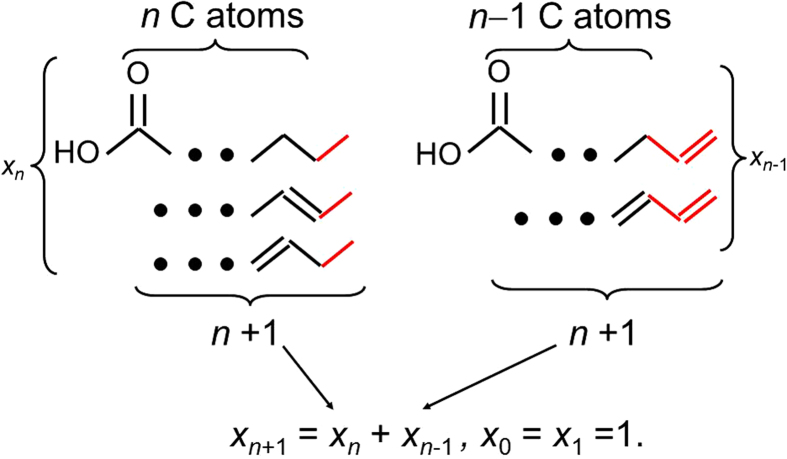
Illustration of the recursive enumeration method. Red lines, bonds added during the procedure. Larger solid dots, variable chain length. Assume we know all *x*_*k*_ from *k* = 1 up to *k* = *n* and wish to calculate *x*_*n*+1_. We can certainly extend the molecule by linking one carbon to the *n*-th carbon by a single bond (left-hand side). Moreover, we can add two carbons to the molecule with *k* = *n*−1, such that carbons *n*−1 and *n* are linked by a single bond and carbons *n* and *n* + 1, by a double bond (right-hand side). Combining the two procedures (starting at *n*−1 and at *n*), we arrive at Eq. (2). There is no overlap between the molecules thus generated because the *x*_*n*_ molecules generated by starting from length *n* have a single bond at the methyl end, while the *x*_*n*−1_ molecules generated by starting from length *n*−1 have a double bond at that end. Moreover, all possibilities of extending the molecules according to the defined rules are covered.

For the concrete case of fatty acids, the recursion is explained in Fig. 2. For example, the three FAs for *n* = 4 are butyric acid (saturated, having its name because of presence in milk and butter), crotonic acid and 3-butenoic acid (both unsaturated) (Fig. 1a). Table 1 shows the Fibonacci numbers for *n *= 1−22. The number series is illustrated in Supplementary Fig. 1.

Besides the binary string problem mentioned above, there are a number of equivalent problems in mathematics. In graph theory, a matching in a graph is a set of edges without common vertices^26^. That edge set corresponds to the double bonds in fatty acids because the latter must not be adjacent (see also Supplementary Fig. 2). The total number of matchings is the Hosoya index, which is particularly easy to compute for linear graphs and then leads to the Fibonacci series (cf. Supplementary Information)^27^.

It is known from number theory^24^ that an explicit formula can be derived from the recursion formula (see also Supplementary Information):


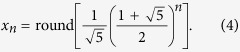


This allows one to calculate the number *x*_*n*_ directly from *n* without the necessity to compute previous numbers first. Note that the explicit formula involves irrational numbers although all Fibonacci numbers are integers. In fact, the ratio 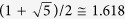 is the legendary Golden section^24^,^25^. Eq. (4) implies that the numbers of FAs show an asymptotically exponential growth with the basis of 1.618. It is understandable that the basis lies between 1 and 2 because there are two possibilities: single bond and double bond, yet the double bonds cannot be adjacent to each other.

An alternative way of proving that the diversity of unmodified fatty acids follows the Fibonacci series is by using a formula derived by Lucas:^24^


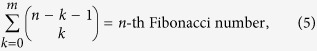


where *m* equals the largest integer that is less or equal to (*n*−1)/2. In this sum, each term can be interpreted such that it describes the number of possibilities of inserting *k* double bonds into a chain length of *n*, in such a way that no double bonds are adjacent to each other nor next to the carboxy end. Due to those constraints, the choice of positions is made out of *n*−*k*−1 positions. That alternative way of computation is more cumbersome than Eq. (4), though, because sums of binomial coefficients need to be computed.

When setting *m* to an arbitrary value less than the value mentioned above, Eq. (5) can be used to compute the number of FAs with at most *m* double bonds. In Table 1, we give the numbers, *q*_*n*_, of FAs with at most six double bonds, by way of example. Two examples of unsaturated FAs with six double bonds are docosahexaenoic acid (22:6), which has cardiovascular-protective properties^8^ and is a structural component of several human organs, and nisinic acid (*all-cis*−6,9,12,15,18,21-tetracosahexaenoic acid, 24:6) in fish. Up to *n* = 14, the series *q*_*n*_ coincides with the Fibonacci numbers *x*_*n*_ because a FA with 14 carbons can harbour up to six carbon-carbon double bonds. For higher *n*, the series *q*_*n*_ grows more slowly, as can also be seen in Supplementary Fig. 1. Any other upper bound *m* on the number of double bonds can be considered in the calculation as well using Eq. (5).

Now, we admit adjacent double bonds, that is, allenic and cumulenic FAs, and denote the number of FAs in this case by *u*_*n*_. Axial stereoisomers are not counted separately here. Both for *n* = 1 and *n* = 2, *u*_*n*_ equals 1 because no double bond can be adjacent to the carboxy group. From *n* = 3 on, the number doubles for each additional carbon atom because there are two possibilities to extend the chain: *u*_*n*+1_ = 2*u*_*n*_. The series reads


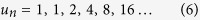


or


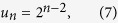


where the first term *u*_1_ needs to be defined separately as 1. In Table 1 and Supplementary Fig. 1, the series is compared to the one defined by Eq. (4).

### Unmodified fatty acids with *cis*- and *trans*-isomers considered separately

When the FAs involve non-terminal double bonds, *cis-* and *trans*-isomers can be distinguished. For example, the *cis*-isomer of crotonic acid is isocrotonic acid (Fig. 1a). This distinction is particularly useful when *cis*- and *trans*-isomers exert different biological functions or different effects on the structure of lipid membranes due to their different molecular shape^15^. Here, we exclude allenic and cumulenic FAs. Special attention needs to be paid to FAs with conjugated double bonds such as in the various isomers of conjugated linoleic acid (18:2) or sorbic acid (6:2). As the corresponding double bonds and the single bonds in between form a π-system, the formal single bonds cannot rotate freely. This gives rise to so-called *s-cis* and *s-trans* isomers. However, the π interaction in these bonds is weaker than in the formal double bonds so that the isomers equilibrate quickly^28^. Therefore, we will only consider *cis*- and *trans*-isomers with respect to double bonds and neglect *s-cis*/*s-trans* isomerism here.

For *n* = 1 and *n* = 2, no double bond is possible. So, the first two numbers in the series are equal to 1. From *n* = 2 on, there are two cases if we add the (*n*+1)-th carbon:

- There is a single bond at position *n*. Then we have two possibilities: Adding a carbon by a single bond or a double bond.
- There is a double bond at position *n*. Then we have again two possibilities: Adding a carbon by a single bond in *cis* configuration or in *trans* configuration.

As in both cases, the number doubles for each additional carbon atom, we obtain the same series as given in Eqs. (6) and (7).

### Modified fatty acids with *cis*- and *trans*-isomers combined

Let us now consider modified FAs, again excluding allenic FAs. Neglecting *cis*-*trans*-isomerism first, we start by allowing oxo groups so that one or several carbons can be linked with oxygen atoms by double bonds. An example is acetoacetic acid (*n* = 4) (Fig. 1b). Biosynthetically, oxidized FAs can be oxylipins (oxidation products of unsaturated FAs) or polyketides^29^,^30^.

Denoting the number of modified FAs by *y*_*n*_, we obtain the recursion formula (for derivation, see Supplementary Information):


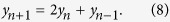


In contrast to the Fibonacci numbers, we have


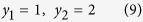


because an oxo group occurs already in glyoxylic acid (Fig. 1b).

Together with these initial conditions, Eq. (8) leads to the series given in the column for *y*_*n*_ in Table 1 and plotted in Supplementary Fig. 1. In mathematics, they are known as the Pell numbers or 2-Fibonacci numbers^24^,^25^,^31^ and obey the explicit formula


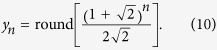


(cf. Supplementary Information), where the basis is the Silver ratio. They represent one instance of generalized Fibonacci numbers given by a linear combination (other than the sum) of the two preceding numbers^32^.

The same series and formula applies to the case where hydroxy groups are allowed instead of oxo groups. In that case, hydroxy groups as part of an enol moiety are not counted because a rapid equilibrium generally favours the corresponding form with an oxo group (keto-enol tautomerism^28^). Similarly, geminal diols are easily converted to the corresponding ketones or aldehydes by loss of one water molecule^28^. Thus, they can be considered equivalent to those molecules. Moreover, we exclude the case where *n* = 1 and a hydroxy group is linked to the only carbon. The corresponding compound, carbonic acid, is an inorganic compound and not considered a FA. When hydroxy groups are included, also different stereoisomers (with *R* and *S* stereocenters) can occur, which are not, however, counted separately here.

Now we further extend this analysis to modified FAs that can contain both oxo and hydroxy groups. We obtain the recursion formula


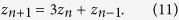


(see Supplementary Information) and the initial conditions


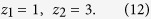


This leads to the series *z*_*n*_ in Table 1 given by the explicit formula


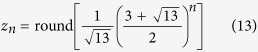


(derivation in Supplementary Information, plot in Supplementary Fig. 1). In mathematics, these numbers are called 3-Fibonacci numbers^24^,^25^.

### Modified fatty acids with *cis*- and *trans*-isomers considered separately

Now, we extend the enumeration procedure for modified FAs by considering *cis*- and *trans*-isomers separately. However, we neglect *R*/*S* stereoisomerism and allenic FAs. We start by allowing oxo groups only or hydroxy groups only and denote the number of modified FAs by *v*_*n*_.

In the Supplementary Information, we derive both a recursion and an explicit formula, which resemble those for the Fibonacci numbers. The new formulas read


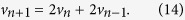



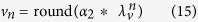


with


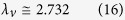


and


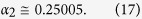


The recursion formula (14) corresponds to another instance of generalized Fibonacci numbers^32^. The initial values can be derived from the first chemical structures,


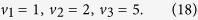


Although Eq. (14) has recursion depth two, three initial values are needed here (see Supplementary Information). From *n* + 1 = 4 on, Eq. (14) generates the number series *v*_n_ given in Table 1. This is also obtained by Eq. (15) for all positive integers *n*.

Now we consider the case where oxo groups and/or hydroxy groups occur and use the symbol *w*_*n*_. In the Supplementary Information, both a recursion formula and an explicit formula are derived:


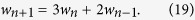



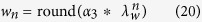


with


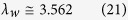


and


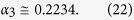


With the relevant initial conditions


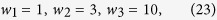


we obtain the number series given in Table 1, column for *w*_*n*_. Eq. (19) holds from *n* + 1 = 4 on.

In the special case of vicinal oxo and hydroxy groups, these groups can swap places due to keto-enol tautomerism involving the corresponding enediol intermediate. This reduces the number of really different FAs. We leave it to future studies to consider this effect in enumerating FAs.

## Discussion

As a starting point for the enumeration of lipid congeners, we deliberately restricted our analysis to certain classes of FAs. Our goal was to derive enumeration formulas for the biochemically most relevant classes of FAs. For example, unmodified FAs with *cis* and *trans* isomers considered separately but neglecting allenic and cumulenic FAs are the basic group of FAs that are usually considered in biochemistry textbooks. We have first derived the number of unbranched, unmodified FAs with different numbers of double bonds to be given by the Fibonacci series when *cis*/*trans* isomerism is neglected. By building the proof on the recursive definition, it is very short and easy to comprehend. Also in enumerating Kekulé structures in chemistry, recursive relations are often used^33^,^34^. In the special case of polyphenanthrenes^33^ and related nanotubes^34^, they lead to Fibonacci numbers as well.

As described above, part of our results can also be derived from the concept of Hosoya index^27^. Nevertheless, to the best of our knowledge, the use of (usual and generalized) Fibonacci numbers for enumerating FAs has not been published before. Extending the present analysis, the Hosoya index can be used also for enumerating branched fatty acids. As shown above, the congeners of fatty acids modified by functional groups can be enumerated by generalized Fibonacci numbers.

Fibonacci numbers are named after Leonardo Pisano, called Fibonacci, although they had been found more than one millennium earlier by scholars in ancient India when studying Sanskrit poems (see Supplementary Information)^35^. In his book “*Liber abaci*” from 1202, Fibonacci derived this series by studying the population dynamics of rabbits. For a sketch of his life in medieval Italy, see ref. 16. The Fibonacci series frequently occurs in biology such as in phyllotaxis and secondary structures of proteins^25^. Interestingly, the Fibonacci sequence is also employed to model X-ray diffraction patterns of films of mixed FA salts^36^.

Although known for a long time, the Fibonacci numbers have lost nothing of their fascination, and more and more fields of application are found. Here, we have shown that fatty acids, which are important molecules in our own body, obey that appealing arithmetics. This finding also applies to analogous classes of terminally monosubstituted hydrocarbons such as aldehydes, alcohols or aliphatic amino acids^37^.

In the case of unmodified FAs, considering *cis*/*trans* isomerism leads to a simple exponential series with the basis of 2. As far as we know, that observation has not been published before either. This number series occurs in the well-known legend of wheat grains on the chessboard^38^. The legend says that a Brahmin called Sessa, the inventor of the game of chess ancestor, chaturanga, was entitled to request a prize from the king. The man asked that on the first square of the chessboard, he would receive one grain of wheat (in some tellings, rice), two on the second one, and so on, doubling the amount each time.

It is somewhat surprising that considering isomerism makes formula (4) for unmodified FAs even easier. For other classes of molecules, it is the other way round. For example, considering stereoisomerism in aliphatic amino acids makes the enumeration more complicated^39^ and so does considering *cis*/*trans* isomerism in the enumeration of modified FAs.

The ratio of two consecutive Fibonacci numbers tends to the Golden section (Supplementary Information). Therefore, starting from an (unmodified) FA of a given length and investing one more carbon atom, an organism can increase the variability of the FA approximately by the Golden section factor, 1.618, or by 2 when *cis*- and *trans*-isomers are counted separately. Furthermore, the fraction of FAs with a terminal single bond is approximately the inverse Golden ratio, 0.618, or 2/3 if *cis*- and *trans*-isomers are counted separately (see Supplementary Information).

An interesting question is why most FAs used in living organisms have chain lengths of 16–22. A biological constraint arises from the thickness of biomembranes, while a physico-chemical constraint is that melting temperatures increase with increasing chain length^40^ so that very long FAs might be too rigid to be used in organisms.

Although interesting, it is beyond the scope of this paper to study in detail which of the theoretically possible FAs are used in living organisms. An impressive number but certainly not all FAs are used in reality. For example, the Fibonacci number *x*_18_ for unmodified FAs is 2,584 and *u*_18_ = 2^16^ = 65,536 (Table 1). Several FAs with *n* = 18 play a role in biology: stearic acid (18:0), oleic acid and its *trans*-isomer elaidic acid (one double bond at position 9), linoleic acid (two double bonds at positions 9 and 12) (Fig. 1a), vaccenic acid (one double bond at position 11) and the various isomers of conjugated linoleic acid (two double bonds, e.g. at positions 9 and 11, or 10 and 12). A few of the latter as well as vaccenic acid occur in cow milk^41^.

The following general observations on naturally occurring FAs are worth noting:

- Practically all chain lengths up to about 35 occur in biological systems^7^.
- Comparing the numbers of naturally occurring FAs with the potential numbers, there appear to be peaks at chain lengths of 16 and 18^1^. However, all shorter lengths occur as well, e.g. capric acid (10:0) occurring in coconut oil and goat milk and inhibiting the yeast-to-hyphae transition of the fungus *Candida albicans*^4^, or *cis*-2-decenoic acid (10:1) made by *Pseudomonas aeruginosa*^42^ and the various medium-length FAs mentioned above.
- FAs rarely involve more than six conjugated double bonds. Therefore, coloured FAs can only rarely be observed in living organisms. The yellowish colour of some adipose tissues comes from carotenoids^43^. Longer conjugated systems show lower stability and, thus, are sensitive to light. It is believed that methyl branches and terminal cyclohexenyl groups, as observed in carotenoids, contribute to increased polyene stability^44^. Two examples of rare modified FAs with extensive conjugated systems are laetiporic acid and its derivative 2-dehydro-3-deoxylaetiporic acid produced by the basidiomycete *Laetiporus sulphureus*, with the former FA being its major orange pigment^45^. They include 10 conjugated carbon-carbon double bonds and one methyl branch each, and the latter even has a further, non-conjugated carbon-carbon double bond.
- Double bonds often occur at a distance of three carbons and then are called homoconjugated. Examples are provided by nisinic acid (24:6, see above) and adrenic acid (*all-cis*-7,10,13,16-docosatetraenoic acid, 22:4).

Beside the academic interest^39^, a promising field of application of this analysis is lipidomics, in which the entirety of a cell’s lipids is studied under different conditions^46^. Lipids and their constituents are most commonly identified by mass spectrometry (MS), and quantification is typically based on comparison of mass-spectrometric ion intensities between individual lipids and suitable standards^47^. Similar to the more advanced proteomics field, the generation of lipidomics data by MS relies on accurate metabolite databases. In analysing the spectra, it is very helpful to know the maximum number of compounds that can potentially appear. Lipid databases are required for the identification in high-throughput and can also guide fragmentation experiments^48^. The formal description presented here may help refine lipid databases and thereby facilitate automated lipid identification as well as in the screening of fungistatic compounds. Moreover, in patent applications related to chemical compounds, it is crucial to know the number of (existing or potential) compounds for which a patent is filed. As outlined in the Supplementary Information, other applications of the presented results concern several aspects of synthetic biology and the understanding of how chemical complexity arose during evolution.

In future studies, it is of interest to derive formulas for larger classes of FAs, using more sophisticated and complex methods. For example, stereoisomers of hydroxylated FAs and the (rarely occurring) FAs involving triple bonds can be studied (for enumeration of amino acids involving triple bonds, see ref. 37). The number of different FAs allowing branching and only carbon-carbon single bonds can be computed in the same way as that of primary alcohols or of aliphatic amino acids only involving single bonds^39^. One suitable method for doing so is based on Pólya’s enumeration theorem^49^. Furthermore, the probability of having double bonds at specific positions (such as ω-3, 6, 9) can be studied.

The recursive definition of Fibonacci and generalized Fibonacci numbers paves the way to list all structures successively in silico. With the above-mentioned coding of FAs as binary strings such as 0001010 and appropriate software, this can be translated into the chemical structures.

## Additional Information

**How to cite this article:** Schuster, S. *et al*. Use of Fibonacci numbers in lipidomics – Enumerating various classes of fatty acids. *Sci. Rep.* **7**, 39821; doi: 10.1038/srep39821 (2017).

**Publisher's note:** Springer Nature remains neutral with regard to jurisdictional claims in published maps and institutional affiliations.

## Supplementary Material

Supplementary Information

## Figures and Tables

**Table 1:** Number of fatty acids (FAs) as a function of chain length *n* for *n* = 1–22.

*n*	*x*_*n*_	*y*_*n*_	*z*_*n*_	*u*_*n*_	*v*_*n*_	*w*_*n*_	*q*_*n*_
**1**	1	1	1	1	1	1	1
**2**	1	2	3	1	2	3	1
**3**	2	5	10	2	5	10	2
**4**	3	12	33	4	14	36	3
**5**	5	29	109	8	38	128	5
**6**	8	70	360	16	104	456	8
**7**	13	169	1,189	32	284	1,624	13
**8**	21	408	3,927	64	776	5,784	21
**9**	34	985	12,970	128	2,120	20,600	34
**10**	55	2,378	42,837	256	5,792	73,368	55
**11**	89	5,741	1.415 × 10^5^	512	15,824	2.613 × 10^5^	89
**12**	144	13,860	4.673 × 10^5^	1,024	43,232	9.306 × 10^5^	144
**13**	233	33,461	1.543 × 10^6^	2,048	1.181 × 10^5^	3.315 × 10^6^	233
**14**	377	80,782	5.097 × 10^6^	4,096	3.227 × 10^5^	1.180 × 10^7^	377
**15**	610	1.950 × 10^5^	1.684 × 10^7^	8,192	8.816 × 10^5^	4.204 × 10^7^	609
**16**	987	4.708 × 10^5^	5.560 × 10^7^	16,384	2.409 × 10^6^	1.497 × 10^8^	979
**17**	1,597	1.137 × 10^6^	1.836 × 10^8^	32,768	6.580 × 10^6^	5.333 × 10^8^	1,560
**18**	2,584	2.744 × 10^6^	6.065 × 10^8^	65,536	1.798 × 10^7^	1.899 × 10^9^	2,455
**19**	4,181	6.625 × 10^6^	2.003 × 10^9^	1.311 × 10^5^	4.912 × 10^7^	6.765 × 10^9^	3,805
**20**	6,765	1.599 × 10^7^	6.616 × 10^9^	2.621 × 10^5^	1.342 × 10^8^	2.409 × 10^10^	5,798
**21**	10,946	3.861 × 10^7^	2.185 × 10^10^	5.243 × 10^5^	3.666 × 10^8^	8.581 × 10^10^	8,679
**22**	17,711	9.322 × 10^7^	7.217 × 10^10^	1.049 × 10^6^	1.002 × 10^9^	3.056 × 10^11^	12,761

The following cases are distinguished: unmodified FAs with *cis-/trans*-isomers combined (*x*_*n*_, Fibonacci numbers), unmodified FAs with *cis-/trans*-isomers considered separately (*u*_*n*_, Sessa numbers), modified FAs that can have either oxo or hydroxy groups with *cis-/trans*-isomers combined (*y*_*n*_, Pell or 2-Fibonacci numbers), modified FAs that can have either oxo or hydroxy groups with *cis-/trans*-isomers considered separately (*v*_*n*_, a form of generalized Fibonacci numbers), modified FAs that can have both oxo and hydroxy groups with *cis-/trans*-isomers combined (*z*_*n*_, 3-Fibonacci numbers), modified FAs that can have both oxo and hydroxy groups with *cis-/trans*-isomers considered separately (*w*_*n*_, another form of generalized Fibonacci numbers), and unmodified FAs with at most six double bonds and *cis-/trans*-isomers combined (*q*_*n*_).

Unmodified FAs with *cis-/trans*-isomers combined and neighbouring double bonds permitted are also described by *u*_n_. A graphical representation is provided in Supplementary Fig. 1.

## References

[b1] Berg J. M., Tymoczko J. L. & Stryer L. Biochemistry, 6^th^ edn., Freeman, New York (2007).

[b2] Degen C., Habermann N., Piegholdt S., Glei M. & Jahreis G. Human colon cell culture models of different transformation stages to assess conjugated linoleic acid and conjugated linolenic acid metabolism: Challenges and chances. Toxicol. In Vitro 26, 985–992 (2012). 22584027 10.1016/j.tiv.2012.05.002

[b3] Mazur P., Meyers H. V., Nakanishi K., El-Zayat A. A. E. & Champe S. P. Structural elucidation of sporogenic fatty acid metabolites from *Aspergillus nidulans*. Tetrahedron Lett. 31, 3837–3840 (1990).

[b4] Shareck J. & Belhumeur P. Modulation of morphogenesis in *Candida albicans* by various small molecules. Eukar. Cell 10, 1004–1012 (2011). 10.1128/EC.05030-11 PMC3165445 21642508

[b5] Dowhan W. Molecular basis for membrane phospholipid diversity: Why are there so many lipids? Annu. Rev. Biochem. 66, 199–232 (1997). 9242906 10.1146/annurev.biochem.66.1.199

[b6] Bostock R. M., Kuc J. A. & Laine R. A. Eicosapentaenoic and arachidonic acids from *Phytophthora infestans* elicit fungitoxic sesquiterpenes in the potato. Science 212, 67–69 (1981). 17747631 10.1126/science.212.4490.67

[b7] Gunstone F. D., Harwood J. L. & Dijkstra A. J. The Lipid Handbook with CD-ROM, 3^rd^ edn., CRC Press, Boca Raton (2007).

[b8] Napier J. A. The production of unusual fatty acids in transgenic plants. Annu. Rev. Plant Biol. 58, 295–319 (2007). 17472567 10.1146/annurev.arplant.58.032806.103811

[b9] Řezanka T. & Sigler K. Odd-numbered very-long-chain fatty acids from the microbial, animal and plant kingdoms. Prog. Lipid Res. 48, 206–238 (2009). 19336244 10.1016/j.plipres.2009.03.003

[b10] Rogalski M. & Carrer H. Engineering plastid fatty acid biosynthesis to improve food quality and biofuel production in higher plants. Plant Biotechnol. J. 9, 554–564 (2011). 21535359 10.1111/j.1467-7652.2011.00621.x

[b11] Dembitsky V. M. & Maoka T. Allenic and cumulenic lipids. Prog. Lipid Res. 46, 328–375 (2007). 17765976 10.1016/j.plipres.2007.07.001

[b12] Aitzetmüller K., Tsevegsüren N. & Vosmann K. A New allenic fatty acid in *Phlomis* (Lamiaceae) seed oil. Fett/Lipid 99, 74–78 (1997).

[b13] Puertollano E., Kolida S. & Yaqoob P. Biological significance of short-chain fatty acid metabolism by the intestinal microbiome. Curr. Opin. Clin. Nutr. Metab. Care 17, 139–144 (2014). 24389673 10.1097/MCO.0000000000000025

[b14] Van Den, Brink D. M. & Wanders R. J. A. Phytanic acid: Production from phytol, its breakdown and role in human disease. Cell. Mol. Life Sci. 63, 1752–1765 (2006). 16799769 10.1007/s00018-005-5463-y PMC11136310

[b15] Funari S. S., Barceló F. & Escribá P. V. Effects of oleic acid and its congeners, elaidic and stearic acids, on the structural properties of phosphatidylethanolamine membranes. J. Lipid Res. 44, 567–575 (2003). 12562874 10.1194/jlr.M200356-JLR200

[b16] Milicević A. & Trinajstić N. Combinatorial enumeration in chemistry. Chem. Modell. 4, 405–469 (2006).

[b17] Faulon J.-L., Visco D. P. Jr. & Roe D. Enumerating molecules. Rev. Comput. Chem. 21, 209–275 (2005).

[b18] Fujita S. Symmetry and combinatorial enumeration in chemistry. Springer, Berlin (1991).

[b19] Fujita S. Adamantane isomers with given symmetries - Systematic enumeration by unit subduced cycle indices. Tetrahedron 46, 365–382 (1990).

[b20] Barnes E. C., Jumpathong J., Lumyong S., Voigt K. & Hertweck C. Daldionin, an unprecedented binaphthyl derivative, and diverse polyketide congeners from a fungal orchid endophyte. Chemistry 22, 4551–4555 (2016). 26880363 10.1002/chem.201504005

[b21] Haranczyk M., Puzyn T. & Ng E. G. On enumeration of congeners of common persistent organic pollutants. Envir. Poll. 158, 2786–2789 (2010). 10.1016/j.envpol.2010.05.011 20619175

[b22] Gutman I. Algebraic structure count of linear phenylenes and their congeners. J. Serb. Chem. Soc. 68, 391–399 (2003).

[b23] Rosen K. H. Discrete Mathematics and its Applications, McGraw-Hill, New York, ch. 8.1 (2012).

[b24] Koshy T. Fibonacci and Lucas Numbers with Applications, Wiley, New York (2001).

[b25] Jean R. J. Phyllotaxis: A Systemic Study in Plant Morphogenesis, Cambridge University Press, Cambridge (1994).

[b26] Diestel R. Graph Theory, Springer, New York (2000).

[b27] Hosoya H. Topological index: A newly proposed quantity characterizing the topological nature of structural isomers of saturated hydrocarbons. Bull. Chem. Soc. Japan 44, 2332–2339 (1971).

[b28] Vollhardt K. P. C. & Schore N. E. Organic Chemistry. Structure and Function, Freeman, New York (2007).

[b29] Blée E. Impact of phyto-oxylipins in plant defense. Trends Plant Sci. 7, 315–322 (2002). 12119169 10.1016/s1360-1385(02)02290-2

[b30] Hertweck C. The biosynthetic logic of polyketide diversity. Angew. Chem. Int. Ed. 48, 4688–4716 (2009). 10.1002/anie.200806121 19514004

[b31] Bicknell M. A primer on the Pell sequence and related sequences. Fibonacci Quart. 13, 345–349 (1975).

[b32] Kalman D. & Mena R. The Fibonacci numbers – exposed. Math. Mag. 76, 167–181 (2003).

[b33] El-Basil S. & Klein D. J. Fibonacci numbers in the topological theory of benzenoid hydrocarbons and related graphs. J. Math. Chem. 3, 1–23 (1989).

[b34] Lukovits I., Graovac A., Kálmán E., Kaptay G., Nagy, P., Nikolić S. . Nanotubes: Number of Kekulé structures and aromaticity. J. Chem. Inf. Comput. Sci. 43, 609–614 (2003). 12653528 10.1021/ci020059k

[b35] Singh P. The so-called Fibonacci numbers in ancient and medieval India. Hist. Math. 12, 229–244 (1985).

[b36] Ganguly P., Sastry M., Choudhury S. & Paranjape D. V. “Turnover” of amphiphile molecules in Langmuir Blodgett films of salts of fatty acids: An X-ray diffraction study. Langmuir 13, 6582–6588 (1997).

[b37] Fichtner M., Voigt K. & Schuster S. The tip and hidden part of the iceberg: Proteinogenic and non-proteinogenic aliphatic amino acids. Biochim. Biophys. Acta Gen. Subj 1861, 3258–3269 (2017). 10.1016/j.bbagen.2016.08.008 27554846

[b38] Horwitz B. & Kling J. The Chess Player, Vol. 1, R. Hastings:, London, (1852).

[b39] Grützmann K., Böcker S. & Schuster S. Combinatorics of aliphatic amino acids. Naturwissenschaften 98, 79–86 (2011). 21120449 10.1007/s00114-010-0743-2

[b40] Knothe G. & Dunn R. O. A comprehensive evaluation of the melting points of fatty acids and esters determined by differential scanning calorimetry. J. Am. Oil Chem. Soc. 86, 843–856 (2009).

[b41] Friesen R. & Innis S. M. *Trans* fatty acids in human milk in Canada declined with the introduction of *trans* fat food labeling. J. Nutr. 136, 2558–2561 (2006). 16988126 10.1093/jn/136.10.2558

[b42] Davies D. G. & Marques C. N. H. A fatty acid messenger is responsible for inducing dispersion in microbial biofilms. J. Bacteriol. 191, 1393–1403 (2009). 19074399 10.1128/JB.01214-08 PMC2648214

[b43] Moloney A. P., Mooney M. T., Kerry J. P., Stanton C. & O’Kiely P. Colour of fat, and colour, fatty acid composition and sensory characteristics of muscle from heifers offered alternative forages to grass silage in a finishing ration. Meat Sci. 95, 608–615 (2013). 23806853 10.1016/j.meatsci.2013.05.030

[b44] Zeeshan M., Sliwka H.-R., Partali V. & Martínez A. The longest polyene. Org. Lett. 14, 5496–5498 (2012). 23072429 10.1021/ol302577d

[b45] Zhou Z.-Y. & Liu J.-K. Pigments of fungi (macromycetes). Nat. Prod. Rep. 27, 1531–1570 (2010). 20694228 10.1039/c004593d

[b46] Brown H. A. Lipidomics: when apocrypha becomes canonical. Curr. Opin. Chem. Biol. 16, 221–226 (2012). 22381642 10.1016/j.cbpa.2012.02.003 PMC3328648

[b47] Han X., Yang K. & Gross R. W. Multi-dimensional mass spectrometry-based shotgun lipidomics and novel strategies for lipidomic analyses. Mass Spectrom. Rev 31, 134–178 (2012). 21755525 10.1002/mas.20342 PMC3259006

[b48] Yang K., Cheng H., Gross R. W. & Han X. Automated lipid identification and quantification by multidimensional mass spectrometry-based shotgun lipidomics, Anal. Chem. 81, 4356–4368 (2009). 19408941 10.1021/ac900241u PMC2728582

[b49] Pólya G. & Read R. C. Combinatorial Enumeration of Groups, Graphs, and Chemical Compounds. Springer, Berlin (2011).

