# Supplementary material for: Use of Fibonacci numbers in lipidomics – Enumerating various classes of fatty acids
Source: Sci Rep. 2017 Jan 10;7:39821. doi: 10.1038/srep39821 (PMC5223158; doi:10.1038/srep39821)
Supplement: Supplementary Information [file srep39821-s1.pdf]

Supplementary Information for:

Use of Fibonacci numbers in lipidomics – Enumerating various classes of fatty acids

Stefan Schuster[*,1], Maximilian Fichtner[1], Severin Sasso[2]

[1]Dept. of Bioinformatics, Friedrich Schiller University,

Ernst-Abbe-Platz 2, 07743 Jena, Germany

[2]Institute of General Botany and Plant Physiology, Friedrich Schiller University,

Dornburger Str. 159, 07743 Jena, Germany

* e-mail: stefan.schu@uni-jena.de

Table of Contents Page

1. Plot of the number of fatty acids as a function of chain length S2

2. Earlier related work S2

3. Explicit formula for the Fibonacci series S4

4. Modified fatty acids with *cis*- and *trans*-isomers combined S6

5. Modified fatty acids with *cis*- and *trans*-isomers considered separately S7

6. The Golden section S11

7. Further biological implications S11

8. Supplementary references S12

1. Plot of the number of fatty acids as a function of chain length

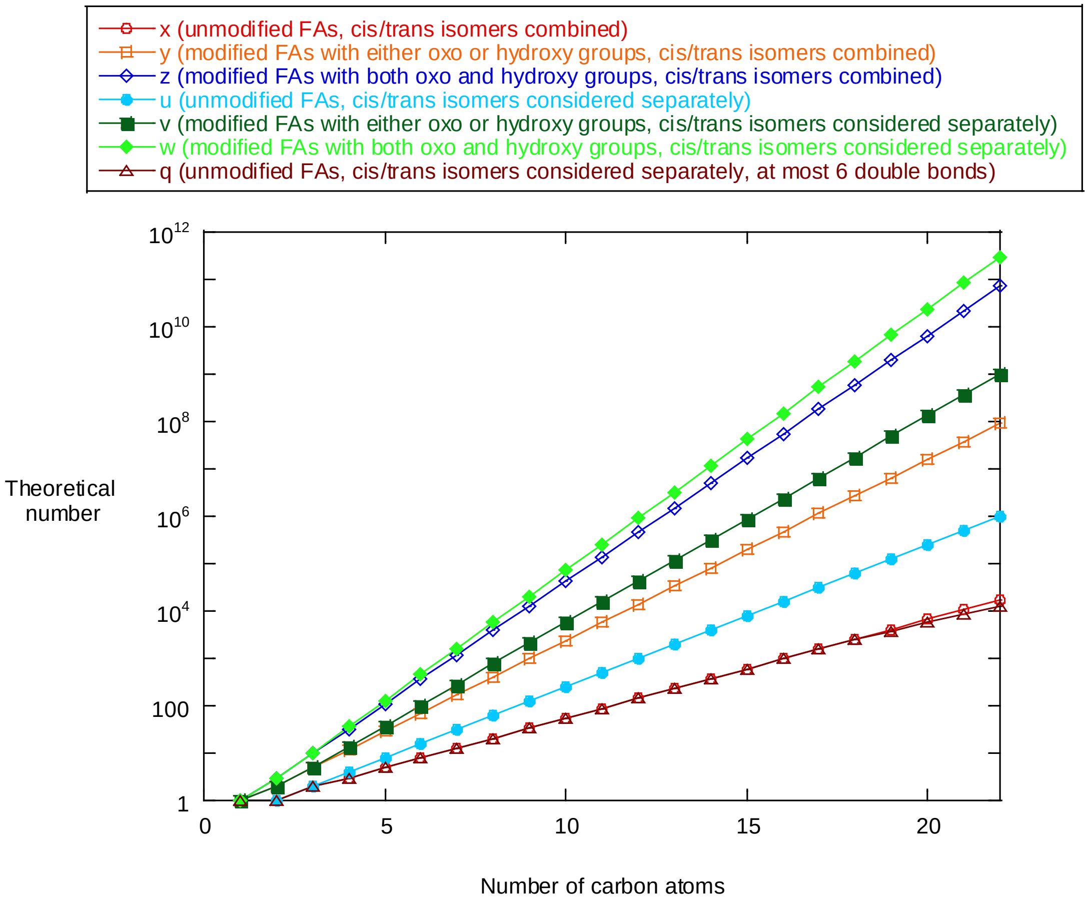

Supplementary Figure 1. Semi-logarithmic plot of the number of fatty acids vs. chain length (for n = 1 to n = 22), cf. Table 1. As all the series (perhaps except q_n, for which this needs to be investigated) grow asymptotically exponentially, the curves in this plot are linear for large n.

2. Earlier related work

In graph theory, a matching in a graph is a subset of edges of the graph without common vertices (Supplementary Fig. 2).[26] That is, these edges should not be adjacent to each other. In FAs, they can be interpreted as double bonds. The total number of matchings is the Hosoya index[23,50] (see also Ref. 16), as illustrated in Supplementary Fig. 2. For the graph shown, five different matchings exist. In FAs, however, the bond next to the carboxy end must be a single bond.

Therefore, the shown matchings are relevant for FAs involving five carbons. In general, the total numbers of matchings in paths of increasing length are given by the Fibonacci series.[27,50,51]
As mentioned in the main text, another equivalent problem is to find the number of binary strings (consisting of 0 and 1 digits) of a given length without adjacent 1 digits.[23] That problem goes back to the study of short ("light") and long ("heavy") syllables (being twice as long as the short syllables) in ancient Sanskrit prosody (about 300-400 BC) and led to the series that is now known as Fibonacci series.[35] If the intervals between two short syllables are coded as 0 and two short syllables linked to a long one are coded as 1, calculating the numbers of patterns of partitioning a given number of beats leads the above-mentioned problem. Also the matchings of an unbranched graph can be encoded as binary strings without adjacent 1 digits (Supplementary Fig. 2). In several recent textbooks on discrete mathematics, as an example of application, the equivalent exercise is given to find a recurrence relation for the number of ways to climb n stairs if one can take one stair or two stairs at a time.[23,52] This can be coded as 0 for one step and (the binary string) 10 for two steps.
In the case of allenic FAs, that is, when adjacent double bonds are allowed, the equivalent string problem is to find all binary strings of a given length (without any restriction). That leads to exponential growth with the basis of two as given in Eq. (7).
As had been mentioned by Hosoya himself, the Hosoya index corresponds to the series of Fibonacci numbers for unbranched paraffins (hydrocarbons) with increasing chain lengths.[27,50] However, he did not mention the relationship to single and double bonds and did not count all unbranched alkanes and alkenes of a given length since he only considered saturated hydrocarbons. Hosoya as well as Randić[53] used the Hosoya index as a molecular descriptor to predict physico-chemical properties such as boiling point, heat of formation, entropy etc. Randić and Pompe[54] considered alkenes in addition, using molecular descriptors to predict molar refraction. Breusch[55] calculated how many constitutional isomers of uniformly polysubstituted, saturated FAs exist, which does not lead to Fibonacci numbers. In none of the cited papers, the counting problem of our main text was considered.

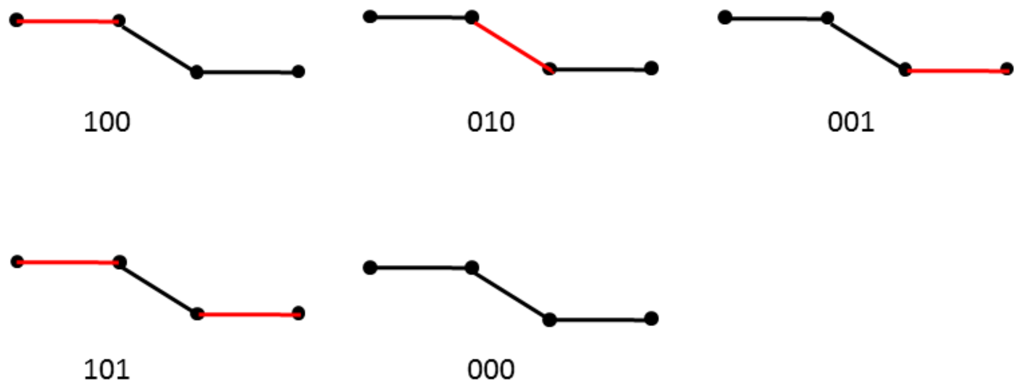

Supplementary Figure 2. Illustration of using the Hosoya index (by coloured graphs) and binary strings for counting fatty acids. Shown are the five matchings of an exemplifying unbranched four-vertex graph, corresponding to the side chain of a FA with five carbon atoms in total. The subsets of edges without common vertices (which can be interpreted as double bonds) are shown in red; the subset is empty in the last case. The total number of matchings (Hosoya index) equals five for the graph shown. For a linear (i.e., unbranched, acyclic) graph, the matchings can be encoded as binary strings without consecutive 1 digits.

3. Explicit formula for the Fibonacci series

Although the derivation of explicit formulas for the Fibonacci series has long been known in number theory[23,24,33,52], we here show it for completeness of the presentation. Eq. (2) is a linear recursion formula. The usual solution procedure is to use an exponential function

$$x_n = \alpha \lambda_x^n \ . \qquad \text{(S1)}$$

In what follows, we specify the basis λ by subscripts corresponding to the symbol used for the series. Substituting Eq. (S1) into the recursion formula (2) leads to the quadratic equation

$$\lambda_x^2 - \lambda_x - 1 = 0 \qquad \text{(S2)}$$

with the solutions

$$\lambda_{x,1/2} = \frac{1 \pm \sqrt{5}}{2} \quad . \tag{S3}$$

The positive solution is the Golden ratio. The explicit formula is obtained by a linear combination of two exponential functions with the two bases given in Eq. (S3).

$$x_n = \alpha_1 \left(\frac{1+\sqrt{5}}{2} \right)^n + \alpha_2 \left(\frac{1-\sqrt{5}}{2} \right)^n \tag{S4}$$

The coefficients α_1 and α_2 are determined by using the initial conditions. It is convenient to start with $n = 0$ rather than $n = 1$ because any number to the power of zero gives unity. x_0 is obtained as $x_0 = x_2 - x_1 = 0$. Thus, Eq. (S4) gives, for $n = 0$ and $n = 1$:

$$0 = \alpha_1 + \alpha_2 \ , \quad 1 = \frac{\alpha_1 + \alpha_2}{2} + \frac{\alpha_1 - \alpha_2}{2}\sqrt{5} \tag{S5a,b}$$

This leads to the Binet formula[23,24,33,52]

$$x_n = \frac{1}{\sqrt{5}} \left(\frac{1+\sqrt{5}}{2} \right)^n - \frac{1}{\sqrt{5}} \left(\frac{1-\sqrt{5}}{2} \right)^n \quad . \tag{S6}$$

Although this formula involves irrational numbers, the resulting numbers are integers. This is because the digits after the period in the two terms of the difference in Eq. (S6) cancel out when calculating the particular x_n.

Eq. (S6) can be simplified due to the observation that the minus solution in Eq. (S3) is less than unity. For $n = 1$, we have

$$-\frac{1}{\sqrt{5}}\left(\frac{1-\sqrt{5}}{2}\right)=0.2764 \tag{S7}$$

while the first term in Eq. (S6) reads

$$\frac{1}{\sqrt{5}}\left(\frac{1+\sqrt{5}}{2}\right)=0.7236\ . \tag{S8}$$

Their sum is $x_1 = 1$. We would obtain the same result by just rounding 0.7236 to an integer value. This procedure also works for any $n > 1$ because the modulus (absolute value) of the negative term in Eq. (S6) is getting smaller and smaller for increasing n, leading to Eq. (4) in the main text.

4. Modified fatty acids with *cis*- and *trans*-isomers combined

Now we derive formulas for FAs with oxo groups. For FAs involving hydroxy groups (but no oxo groups), the calculation is the same. Since carbons are of valence four, oxo groups adjacent to carbon-carbon double bonds (so-called ketenes) would only be possible at the methyl end of the side chain, but will not be considered here due to the instability of this arrangement. The recursion procedure outlined in the main text and illustrated in Fig. 2 can be adapted by saying that not only a methyl group but, alternatively, a -CH=O group can be appended to the n-th carbon atom via a carbon-carbon single bond. To include a double bond between two carbons, we again start at the FA with $n-1$ carbons, but then cannot add an oxo group to this carbon-carbon double bond. This leads to the recursion formula (8) in the main text, which together with the initial values given in Eq. (9), defines the Pell numbers. That series can be found in the Online Encyclopedia of Integer Sequences at www.oeis.org by searching for index A000129. Using again an exponential function ansatz (Eq. (S1)) leads to the quadratic equation

$$\lambda_y^2 - 2\lambda_y - 1 = 0 \tag{S9}$$

with the solution

$$\lambda_{y,1/2} = 1 \pm \sqrt{2} \ . \tag{S10}$$

The positive solution is the Silver ratio. Taking into account the initial conditions, we obtain the explicit formula[24,31]

$$y_n = \frac{\left(1+\sqrt{2}\right)^n - \left(1-\sqrt{2}\right)^n}{2\sqrt{2}} \tag{S11}$$

Again, the first term is always near to the correct integer value. Thus, we can simplify this to Eq. (10) given in the main text.

In the case of FAs that can have both oxo and hydroxy groups, the recursion works as follows. A methyl group, oxo group or hydroxy group can be appended to the *n*-th carbon atom via a carbon-carbon single bond. To include a double bond between two carbons, we again start at the FA with *n*–1 carbons, but then can only add a further methyl group. This leads to the recursion formula (11), which together with Eq. (12) defines the 3-Fibonacci numbers. That series can be found at www.oeis.org (index A006190).

Analogously as above, the following explicit equation for 3-Fibonacci numbers and, thus, for FAs with two types of possible functional side groups (e.g. oxo and hydroxy) is derived:

$$z_n = \frac{1}{\sqrt{13}}\left(\frac{3+\sqrt{13}}{2}\right)^n - \frac{1}{\sqrt{13}}\left(\frac{3-\sqrt{13}}{2}\right)^n \ , \tag{S12}$$

which can be simplified to Eq. (13) in the main text. The positive basis is the Bronze ratio.

5. Modified fatty acids with *cis*- and *trans*-isomers considered separately

We first derive a recursion formula for FAs that can contain either oxo or hydroxy groups. Here, we exemplify this by hydroxy groups and illustrate it in Supplementary Fig. 3. Assume we know v_i for all i=1 to n. Now we want to determine v_{n+1}. Starting from a FA with n carbons, we can add

either a methyl group by a single bond or a hydroxymethyl group (-CH_2OH) by a single bond. If the carbons n–1 and n are linked by a single bond, then there is only one possible configuration for the additional single bond. If the carbons n–1 and n are linked by a double bond, then we add the additional single bond in one of the possible configurations, say *cis*. Thus, the transition from n to n+1 gives a term $2v_n$ in the recursion (Supplementary Fig. 3, left-hand side).

Before generating the additional structures, we define the following quantities. For each integer i>1, we note that there are as many molecules involving i carbons and ending with a single bond and a methyl group as there are molecules ending with a single bond and a -CH_2OH group. We denote this number by a_i. The remaining number of FAs with i carbons, which end with a =CH_2 group, is denoted by b_i. Thus, $v_i = 2a_i + b_i$.

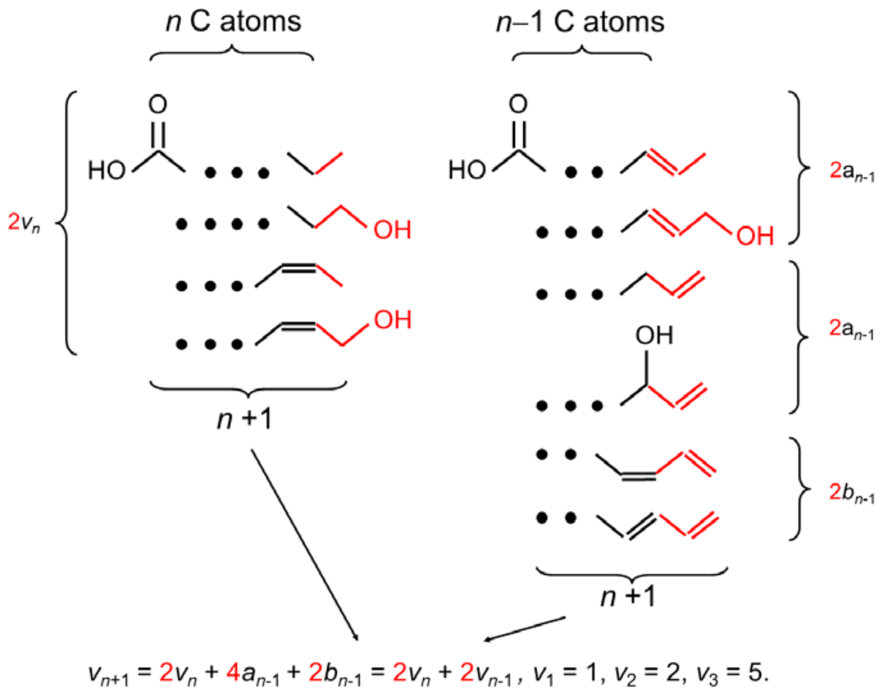

Supplementary Figure 3. Illustration of the recursive enumeration method for modified fatty acids that can contain hydroxy groups, with *cis*- and *trans*-isomers considered separately. Possibly occurring additional hydroxy groups in the two structures on the upper left are not shown. Red lines, bonds added during the procedure. Larger solid dots, variable chain length. Further explanations, see text.

We now put $i=n-1$. We can extend the a_{n-1} molecules ending with a single bond and a methyl group by (i) a double bond and a single bond to a methyl group or (ii) a double bond and a single bond to a -CH_2OH group. In both cases, we choose the *trans* configuration. This complements the molecules in *cis* configuration generated above and adds a term $2a_{n-1}$ to the recursion (Supplementary Fig. 3, right-hand side).

Moreover, to *all* $2a_{n-1}$ FAs with $n-1$ carbons, we can add a single bond and a double bond, generating two new ends like that: -CH_2-CH=CH_2 and -CHOH-CH=CH_2. This adds a term $2a_{n-1}$ to the recursion once more.

To the b_{n-1} FAs with $n-1$ carbons ending with a double bond, we add a vinyl group (-CH=CH_2) in two ways: so that the double bond between carbons n-2 and n-1 is in *cis* or in *trans*-configuration. Thus, also the number of the molecules ending with a double bond should be doubled in the recursion. In this way, we generate all possible extended structures (without overlap between the generated structures) and obtain the recursion formula (14).

Special attention must be paid to the initial values. For n=1, there is only one possibility for the side chain: one single hydrogen, corresponding to formic acid. For n=2, v_2=2 because the side chain can consist of a methyl group or a -CH_2OH group. For n+1=3, we cannot yet apply recursion (14) because we excluded the molecule involving one carbon only and a hydroxyl group (carbonic acid). Thus, only the factor 1 rather than 2 should be assigned to v_1 in the recursion: $v_3 = 2\ v_2 + v_1 = 2*2 + 1 = 5$. From n+1=4 on, we can apply recursion (14). This gives rise to the number series 1, 2, 5, 14, 38, 104, … given in Table 1 (index A052945 at www.oeis.org). The quadratic equation leads to the basis λ_v = 1+SQRT(3) = 2.732… for the explicit formula (15).

A simple way of writing an explicit formula is by deriving a coefficient to λ_v^n and rounding. This can be done by dividing a sufficiently large v_n (e.g. v_{10}) by λ_v^n. Thus, we obtain the coefficient $\alpha_2 \cong 0.25005$, leading to the explicit formula (15). For n = 1, for example, the term in parentheses in Eq. (15) is 0.6831, which correctly gives v_1 = 1 upon rounding.

In an analogous way, we can derive equations for the case where both oxo and hydroxy groups can occur. We again proceed in a recursive way. Starting from a FA with n carbons, we can add either a methyl group or a -CH_2OH group or a -CH=O group, each by a single bond. If the carbons $n-1$ and n are linked by a single bond, then there is only one possible configuration for

the additional single bond. If the carbons n–1 and n are linked by a double bond, then we arrange the additional single bond so that the double bond is in one of the possible configurations, say *cis*. Thus, the transition from n to n+1 gives a term 3 w_n in the recursion.

For each integer i>1, we note that there are as many molecules involving i carbons and ending with a single bond and a methyl group as there are molecules ending with a single bond and a -CH_2OH group and also as there are molecules ending with a single bond and a -CH=O group. We denote this number by c_i. The remaining number of FAs with i carbons, which end with a double bond, is denoted by d_i. Thus, $w_i = 3\ c_i + d_i$. We now put i=n–1. We can extend the c_{n-1} molecules ending with a single bond and a methyl group by (i) a double bond and a single bond to a methyl group, (ii) a double bond and a single bond to a -CH_2OH group or (iii) a double bond and a single bond to a -CH=O group. In both cases, we choose the *trans* configuration. This complements the molecules in *cis* configuration generated above and adds a term 3 c_{n-1} to the recursion.

Moreover, to all 3 c_{n-1} FAs with n–1 carbons, we can add a single bond and a double bond, generating three new ends like that: -CH_2-CH=CH_2 and -CHOH-CH=CH_2 and –C=O-CH=CH_2. This adds a term 3 c_{n-1} to the recursion once more.

To the d_{n-1} FAs with n–1 carbons ending with a double bond, we add a vinyl group (-CH=CH_2) in two ways: so that the double bond between carbons n-2 and n-1 is in *cis* or in *trans*-configuration. Thus, also the number of the molecules ending with a double bond should be doubled in the recursion. In this way, we generate all possible extended structures and obtain the recursion formula (19).

Special attention must be paid to the initial values. For n=1, there is formic acid only. For n=2, w_2=3 because the side chain can consist of a methyl group, a -CH_2OH group or -CH=O group. For n+1=3, we cannot yet apply recursion (19) because we excluded the molecule involving one carbon only and a hydroxy group (carbonic acid); a (second) oxo group is impossible anyway. Thus, only a value of 1 rather than 2 should be assigned to w_1 in the recursion: $w_3 = 3\ w_2 + w_1$ = 3*3 + 1 = 10. From n+1=4 on, we can apply recursion (19). This gives rise to the number series w_n given in Table 1. The quadratic equation leads to the basis λ_w = 3/2+SQRT(17)/2 = 3.56155… for the explicit formula. With an appropriate coefficient, the explicit formula (20) is obtained.

6. The Golden section

It is known from mathematics that the ratio of two consecutive Fibonacci numbers tends to the Golden section. This can be shown by substituting Eq. (4) into x_{n+1}/x_n. As the difference to the rounded value is getting smaller and smaller, it can be neglected in that ratio for large n. This leads to the following observation. In the construction procedure of the FAs shown in Fig. 2, which we used to derive the recursion formula (2), we added a terminal double bond by starting from the FAs with n–1 carbons, while we added a terminal single bond by starting from the FAs with n carbons. As also x_n/x_{n-1} tends to the Golden section, the ratio of the numbers of FAs with a terminal single bond and a terminal double bond (for a given chain length) approximately equals the Golden section, 1.618, and converges to that number with increasing chain length. The inverse ratio is 0.618. Note that the digits after the period are the same, which is one of the striking properties of the Golden section. The properties of the Golden section then imply that the fraction of FAs with a terminal single bond (compared to all FAs of the considered chain length) tends to 0.618.

An analogous calculation can be done in the case where *cis*- and *trans*-isomers are counted separately. Let d_n denote the number of FAs of length n with a terminal double bond. Thus, $u_n – d_n$ is the number of FAs with a terminal single bond. The ratio d_n/u_n shows a non-trivial pattern: For n = 1–7, for example, it follows the series 0, 0, 1/2, ¼, 3/8, 5/16, 9/32. We denote the limit value of this series by γ. In the transition from n to n+1, a double bond can only be added to a FA with a terminal single bond, and only in one configuration. Thus, $d_{n+1} = u_n – d_n$. Moreover, due to the recursion for u_n, we have

$$d_{n+1}/u_{n+1} = \gamma = (u_n - d_n)/(2u_n) = (1 - \gamma)/2 \qquad \text{(S13)}$$

This leads immediately to γ = 1/3. Thus, the fraction of FAs with a terminal double or single bond tends to 1/3 or 2/3, respectively, when *cis*-/*trans*-isomerism is considered.

7. Further biological implications

A further application of this work is to estimate the time necessary to perform, in the laboratory, the chemical synthesis of all FAs of a certain length. This could also be of interest in synthetic

biology, which is aimed at constructing systems (e.g. metabolic pathways) that have never been present within living organisms.[56] Such engineered systems could produce FAs not found before. Our analysis can also help in understanding principles of evolution including prebiotic evolution. In living organisms, only relatively few building blocks out of an enormous theoretical number are used. Out of more than 100 chemical elements, only six are mainly used: C, H, O, N, S and P. Only four nucleobases appear in the DNA; proteins are built from a limited set of amino acids. For example, the number of proteinogenic aliphatic amino acids is exceeded by far by the number of naturally occurring non-proteinogenic versions and even more so by the theoretically possible structures, for which a recursion formula can be given.[39] Biological complexity then arises by a versatile combination of a few building blocks.

As for FAs, a strikingly high number occurs in nature, but much less than the number of theoretically conceivable structures. The realized number might be that high because FAs are, in a sense, building blocks and polymers at the same time. Due to the synthesis by assembling two-carbon units, which also applies to many polyketides, many possibilities arise in inserting double bonds, hydroxy groups and other functional groups. An additional source of complexity is the combination of FAs into phospholipids and triglycerides.

Besides the more widely known “RNA world” scenario for prebiotic evolution[56], some authors have put forward the idea of a lipid world where the first self-replicating unit was a lipid vesicle or micelle.[57] Most likely, lipid diversity has affected the course of prebiotic and biotic evolution many times.

8. Supplementary references

50. Hosoya, H. Topological index and Fibonacci numbers with relation to chemistry. *Fibonacci Quart.* **11**, 255-266 (1973).

51. Došlić, T. & Litz, M.S. Matchings and independent sets in polyphenylene chains. *MATCH Commun. Math. Comput. Chem.* **67**, 313-330 (2012).

52. Matoušek, J. & Nešetřil: *Invitation to Discrete Mathematics*. Oxford University Press, Oxford (2003).

53. Randić, M. Wiener-Hosoya index - a novel graph theoretical molecular descriptor. *J. Chem. Inf. Comput. Sci.* **44**, 373-377 (2004).
54. Randić, M. & Pompe, M. On characterization of the CC double bond in alkenes. *SAR and QSAR Environ. Res.* **10**, 451-471 (1999).
55. Breusch, F. L. Anzahl der Isomeren von polysubstituierten Fettsäuren. *Fette, Seifen, Anstrichm.* **72**, 1-6 (1970).
56. Szostak, J. W., Bartel, D. P. & Luisi, P. L. Synthesizing life. *Nature* **409**, 387-390 (2001).
57. Segrè, D., Ben-Eli, D., Deamer, D. & Lancet, D. The lipid world. *Origins Life Evol. B.* **31**, 119-145 (2001).
